# Studying the cation dependence of CO_2_ reduction intermediates at Cu by *in situ* VSFG spectroscopy†

**DOI:** 10.1039/d3sc05295h

**Published:** 2024-01-17

**Authors:** Liam C. Banerji, Hansaem Jang, Adrian M. Gardner, Alexander J. Cowan

**Affiliations:** a Department of Chemistry, Stephenson Institute for Renewable Energy, University of Liverpool Liverpool UK acowan@liverpool.ac.uk; b Early Career Laser Laboratory, University of Liverpool Liverpool UK

## Abstract

The nature of the electrolyte cation is known to have a significant impact on electrochemical reduction of CO_2_ at catalyst|electrolyte interfaces. An understanding of the underlying mechanism responsible for catalytic enhancement as the alkali metal cation group is descended is key to guide catalyst development. Here, we use *in situ* vibrational sum frequency generation (VSFG) spectroscopy to monitor changes in the binding modes of the CO intermediate at the electrochemical interface of a polycrystalline Cu electrode during CO_2_ reduction as the electrolyte cation is varied. A CO_bridge_ mode is observed only when using Cs^+^, a cation that is known to facilitate CO_2_ reduction on Cu, supporting the proposed involvement of CO_bridge_ sites in CO coupling mechanisms during CO_2_ reduction. *Ex situ* measurements show that the cation dependent CO_bridge_ modes correlate with morphological changes of the Cu surface.

## Abstract 2

The nature of the electrolyte cation has a significant impact on electrochemical reduction of CO_2_ at catalyst|electrolyte interfaces. VSFG spectroscopy shows that the presence of Cs^+^ correlates with CO_(bridge)_ formation, indicating a role in C_2_ product formation.

## Graphical abstract

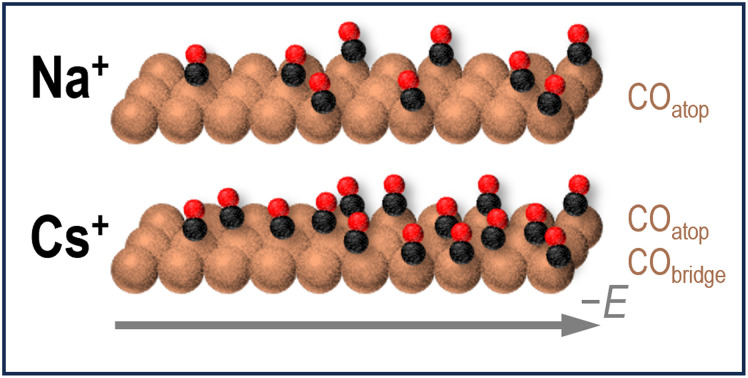

## Introduction

Anthropogenic climate change has led to the urgent requirement for sustainable carbon cycles to be developed. The use of CO_2_ through capture and conversion technologies has been identified as a promising way to generate chemical feedstocks and fuels.^1^ Electrochemical CO_2_ reduction (eCO_2_R) has been studied extensively at a range of metal surfaces; however there is still an incomplete mechanistic understanding of the eCO_2_R reaction.^2^ Cu has been identified as the only heterogeneous, monometallic catalyst to be able to reduce CO_2_ to products that require more than two electrons in the reaction pathway in appreciable quantities, including many short-chain oxygenates and hydrocarbons (C_2+_ products).^3–5^ This is hypothesised to be due to optimal binding energies of crucial intermediates on Cu surfaces, shown by density functional theory (DFT) studies.^6^

The nature of the catalyst is only one of the many factors which influence the performance of eCO_2_R; cell architecture and electrochemical environment both also play major roles. The species of the electrolyte has been shown to dramatically alter the product distribution of the reaction at Au,^7–9^ Ag^10–12^ and Cu.^11–17^ Specifically, the alkali metal cation species present in the electrolyte has a profound effect on the faradaic efficiency (FE) towards target carbon products. When Cu is the electrode, the C_2_/C_1_ product ratio is also reported to increase in the order Li^+^ < Na^+^ < K^+^ < Rb^+^ < Cs^+^. The dependence of product distribution on cations has also been detected in zero-gap cathode structures, where cation cross-over from a liquid analyte through an anion exchange membrane occurred.^18^ The hypotheses for the mechanism of these catalytic enhancements are widely debated within the community; the three predominant arguments can be summarised as (i) the p*K*_a_ of the water molecules in the hydration sphere of the cation at the cathode decreases as the alkali metal group is descended from Li^+^ to Cs^+^ increasing the available CO_2_ by minimising conversion to bicarbonate locally.^12,19^ (ii) An increase in the potential drop across the outer Helmholtz plane (OHP), and hence local electric field, due to the smaller hydrated radius going from Li^+^ to Cs^+^.^20^ The increased electric field can benefit eCO_2_R kinetics *via* a number of mechanisms, including reducing the activation barrier for charge transfer and through the stabilisation of reaction intermediates.^20^ Finally (iii) stabilisation of intermediates through specific cation/reaction intermediate interactions has been proposed, impacting on both CO_2_ and CO binding strengths and the CO coupling reactions^11,21–24^ and supporting this conclusion is the recent demonstration that eCO_2_R does not occur in rigorously prepared solutions where the cation has been excluded.^9^

Vibrational spectroscopies^19,25–29^ such as surface enhanced infrared absorption spectroscopy (SEIRAS), surface enhanced Raman spectroscopy (SERS) and shell isolated nanoparticle enhanced Raman spectroscopy (SHINERS) have been used extensively to study the electrode–electrolyte interface during eCO_2_R and to explore the role of cations. SEIRAS measurements have studied bicarbonate at an operating electrode providing evidence for the role of the cation in pH buffering during eCO_2_R.^19^ The potential dependent (often labelled vibrational Stark) tuning rates of vibrational probes (such as CO) at Au,^28,29^ Cu^21^ and Pt^26^ have also been reported and these can provide an indication of local field strength. Such studies show that the field strength correlates with an increased interfacial cation concentration and a decreased hydrated cation radius. However, it is important to note that in many cases only a single metal–CO binding site is discussed. Furthermore a recent study of a Au electrode measured the field strengths arising at the electrode from the applied potential and found these to be relatively small when compared to the solvation induced Onsager reaction field, which is induced by the cation through direct interaction with surface CO.^29^

Although SEIRAS and SERS have provided important insights into the eCO_2_R reaction, both techniques require surface modification of the electrode. This is typically roughening, or in some cases deposition of the catalysts onto a suitably roughened support, in order to gain sufficient enhancement factors to enable identification of surface species. A concern is that samples suitable for SEIRAS/SERS can show significantly different catalytic activities to the polycrystalline electrode surfaces that are routinely employed in eCO_2_R and this can be correlated to differences in CO binding sites.^25^ CO has been identified linearly bound atop (CO_atop_) and at bridging sites (CO_bridge_) by attenuated total reflection (ATR) FTIR and also SERS spectroscopies.^30–40^ Whilst CO_bridge_ has been considered to be irreversibly bound and not active on the eCO_2_R pathway,^33^ recent works have proposed that the presence of CO_bridge_ leads to an increase in C_2_ products,^30,34,36,37^ with DFT calculations suggesting that CO–CO coupling may be more facile between CO_atop_ and CO_bridge_.^36^ Interestingly, a preliminary ATR-FTIR study noted that CO_bridge_ sites are present in Cs^+^ containing electrolytes^21^ which are proposed to promote C_2_ formation and a similar observation was made by Raman spectroscopy.^35^ However, as highlighted in a recent review,^41^ there is a need for improved understanding of the nature of surface intermediates during eCO_2_R at Cu and their dependence on the reaction conditions. Here we use VSFG spectroscopy to study *ν*(CO) at polycrystalline Cu (pc-Cu) electrodes to identify relative populations of reaction intermediates and their evolution in the presence of different electrolyte salts. Three alkali metal cations have been chosen for analysis: K^+^, which is the most commonly used electrolyte cation for eCO_2_R, Cs^+^, which has been shown to give the greatest enhancement for C_2+_ products, and Na^+^, which shows poorer eCO_2_R performance than K^+^ whilst maintaining appreciable levels of C-based products. The ability of VSFG to study catalyst|electrolyte interfaces without the need for modifications, as required in the aforementioned spectroelectrochemical studies, which can fundamentally alter the electrodes activity, makes it an important tool to assess the mechanisms occurring on the pc-Cu electrodes routinely employed for eCO_2_R.

## Results and discussion

**Fig. 1:**
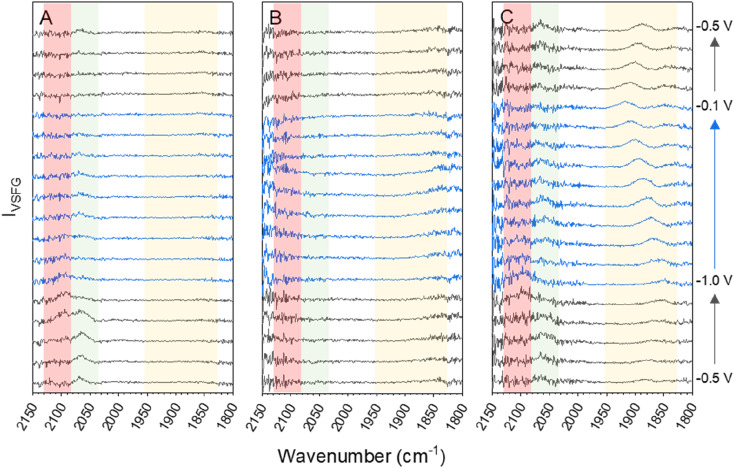
VSFG spectra showing CO modes on pc-Cu across the studied potential window CO_2_-purged 0.5 M (a) NaHCO_3_, (b) KHCO_3_ and (c) CsHCO_3_ during sequential potential measurements (−0.5 → −1.0 → −0.1 → −0.5 V *vs.* Ag^+^ in 0.1 V increments). In each panel the data for the reverse steps (−1.0 → −0.1 V) is in blue to guide the eye. The red, green and yellow shaded regions highlight the spectral range where bands are assignable to *ν*(CO_atop(defect)_), *ν*(CO_atop(terrace)_) and *ν*(CO_bridge_) sites respectively.

The data for the *in situ* VSFG experiments on a pc-Cu electrode in 0.5 M Na^+^, K^+^ or Cs^+^ bicarbonate electrolytes purged with CO_2_ at a range of applied potentials is shown in Fig. 1. Full experimental details are provided in the Methods section and the experiment design is shown in Fig. S1.† The potential is held for 150 seconds with the VSFG spectrum collected for 120 s, following a 30 s equilibration period. The potential steps progress in the order −0.5 → −1.0 → −0.1 → −0.5 V *vs.* Ag^+^ in 0.1 V increments. Progressing the potential steps in this order ensures that the Cu surface is fully reduced at the start of the experiment. All potentials from this point onwards will be referenced against the Ag^+^ pseudo reference electrode and the choice of reference electrode is discussed in ESI Note 1.† The potential window was chosen following linear sweep measurements of the same electrode which showed that the catalytic onset potential under CO_2_ was *ca.* −0.8 V for all of the electrolytes (Fig. S2†). As discussed in ESI Note 2,† VSFG spectra are recorded with a short time delay (*ca.* 0.7 ps) between the mIR pulse (centred at approximately 1920 cm^−1^, 170 fs duration) and a time-asymmetric nIR picosecond laser pulse in order to minimise the non-resonant SFG response that can otherwise dominate the VSFG spectrum.^42^ Spectra are also normalised to the intensity of the mIR profile as discussed in ESI Note 3.†

### The CO binding site on Cu is dependent on the cation type and applied potential

**Fig. 2:**
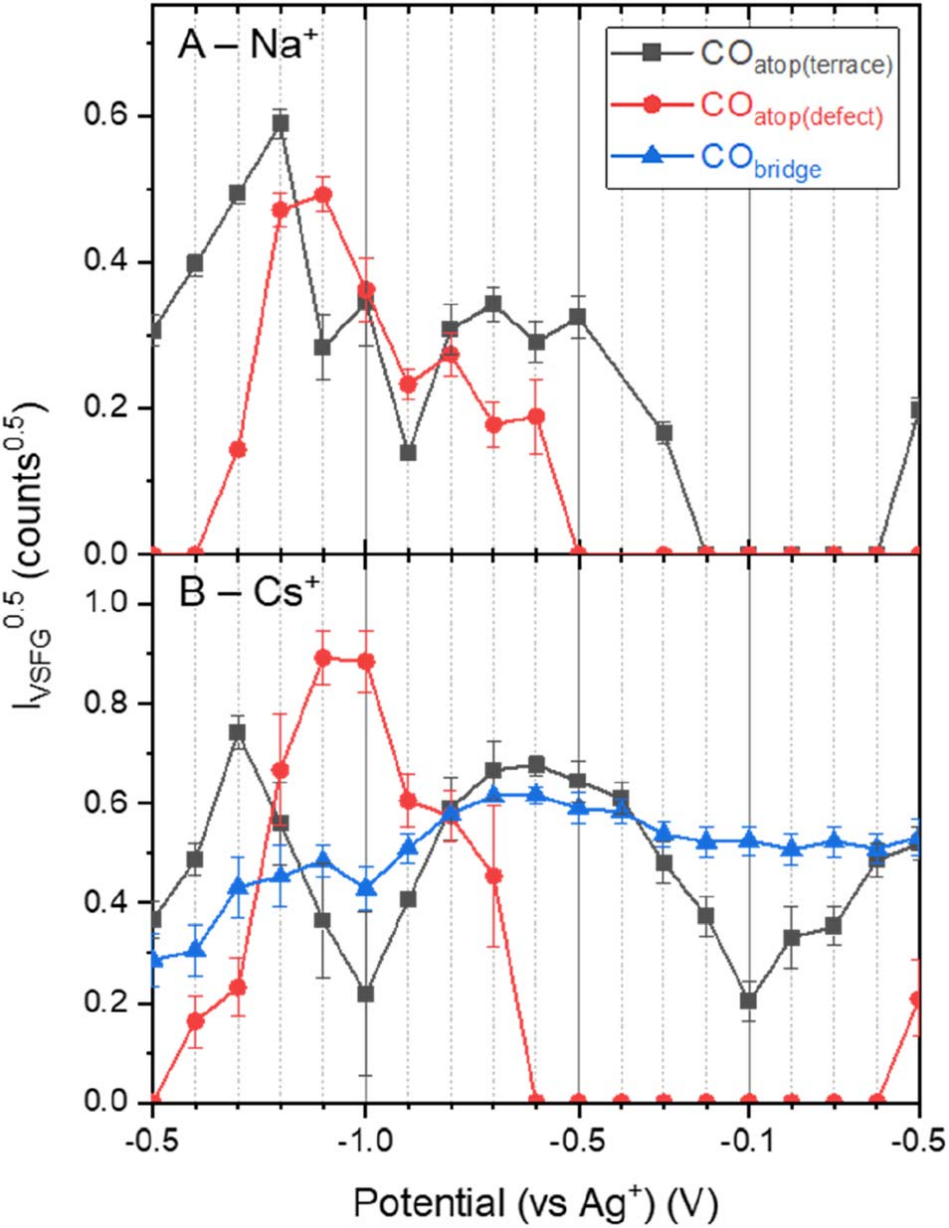
Square root of the area of the different VSFG measured CO modes (*I*_VSFG_^0.5^) as an estimate of surface concentration on Cu as a function of applied potential and electrolyte salt (a) NaHCO_3_, (b) CsHCO_3_, during eCO_2_R. Values of 0 counts indicate that the signal : noise for the peak was insufficient to enable convergence for the fit. Populations could not be estimated for the KHCO_3_ system in the absence of *I*_VSFG_ for this data set.

Fig. 1 shows that *ν*(CO) are detected by *in situ* VSFG spectroscopy on the pc-Cu electrode during eCO_2_R but the nature and presence of *ν*(CO) is dependent upon the choice of electrolyte cation. In the presence of Na^+^, a single *ν*(CO) at approximately 2062 cm^−1^ is present at −0.5 V. A second band at 2094 cm^−1^ grows in at −0.8 V and follows to become the dominant band by −0.9 V. As discussed below the exact positions of the *ν*(CO) depends on the applied potential. The presence of adsorbed CO at −0.5 V, positive of the anticipated onset potential for eCO_2_R (Fig. S2†), is due to the production of CO during prior measurements on the cell (ESI Note 2†). The 2062 and 2094 cm^−1^ bands can be assigned to CO linearly bound at terraced (CO_atop(terrace)_) and defect (CO_atop(defect)_) sites, respectively.^25,27,43,44^ VSFG band intensities (*I*_VSFG_) recorded by homodyne detection scale quadratically with number density and are also dependent on the orientation of the vibrational mode and ordering at the surface.^45^ Here we use (*I*_VSFG_)^0.5^ as a way to estimate the relative populations of the different CO binding modes, Fig. 2, with *I*_VSFG_ determined from the peak area obtained by Lorentzian peak fitting. As shown in Fig. 2, when using NaHCO_3_ CO_atop(terrace)_ accumulates between −0.5 and −0.8 V, whilst CO accumulates at defect sites between −0.7 and −0.9 V. It is reported that CO_2_ reduction to CO occurs preferentially at defect sites, formed following the reduction of surface oxides, and the increase in population of CO_atop(defect)_ at potentials around where eCO_2_R onsets (*ca.* −0.8 V, Fig. S2† and 2) is in line with this proposal.^46^ However, we note that defect formation on Cu is known to be sensitive to CO coverage and we cannot rule out that the potential dependence of CO_atop(defect)_ is due to the potential dependent formation of the binding site or relative potential dependent stability of the binding sites, and this is discussed further below.

**Fig. 3:**
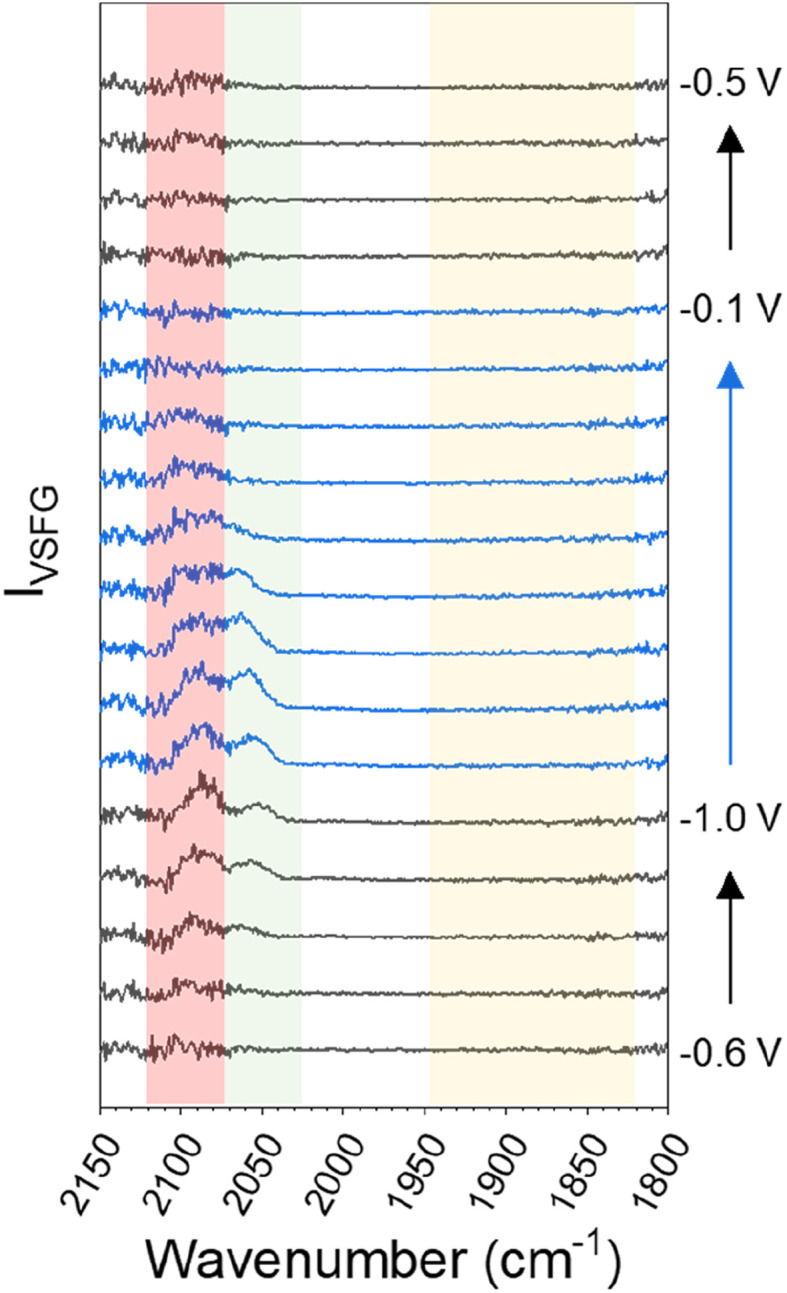
VSFG spectra showing CO modes on Cu across the studied potential window in CO-purged 0.5 M CsHCO_3_ at the potentials indicated. The red and green shaded regions highlight the spectral range where bands are assignable to *ν*(CO_atop(defect)_) and *ν*(CO_atop(terrace)_) sites respectively. A background subtraction using the first −0.5 V spectrum (where no CO modes are observable) has been performed on all spectra to remove interference in the CO_atop_ region by supercontinuum generation, shown in Fig. S7 and discussed in ESI Note 3.†

When CsHCO_3_ is used as the electrolyte during eCO_2_R the two VSFG bands assigned to CO_atop(terrace)_ and CO_atop(defect)_ at approximately 2066 and 2094 cm^−1^ respectively are present, alongside an additional band at 1887 cm^−1^ (at −0.5 V), Fig. 1C. The frequency of the band at 1887 cm^−1^ matches that previously assigned to CO_bridge_ binding at Cu.^30,33,34,38,39,41,47^ Past studies have proposed that bridge bound CO is electrochemically stable^30,33,38,47^ and we also observe that the band at 1887 cm^−1^ at −0.5 V is stable across a wide potential window (−0.1 to −1.0 V, Fig. 2A and S5†) and that CO accumulates at this site during the experiment. The observation that CO_bridge_ is present when Cs^+^ is used as an electrolyte but not Na^+^ is notable. When the VSFG experiment in CsHCO_3_ is repeated under CO instead of CO_2_, the CO_bridge_ species is not observed, Fig. 3. This demonstrates that the generation of and/or occupancy of the CO_bridge_ site relies upon the specific local conditions generated during eCO_2_R. Both the CO coverage and concentration in the electrolyte have also been shown to be important factors in the reconstruction of Cu surfaces,^27,48^ which may explain the evolution of different binding motifs when compared to the VSFG data during eCO_2_R.

Fig. 1B shows that VSFG experiments in KHCO_3_ have very low *I*_VSFG_ bands only. Previously a lack of observation of *ν*(CO_atop_) bands by VSFG on pc-Cu has been attributed to the presence of a disordered CO layer on the electrode.^49^ It is well established that CO can be measured at Cu electrodes in KHCO_3_ by linear vibrational spectroscopies (*e.g.* SEIRAS, Raman).^27,36,37,44,50–56^ The lack of detected Cu–CO by VSFG during eCO_2_R using a KHCO_3_ is therefore proposed to be due to disorder of the Cu–CO surface. Overall, it is clear that there is a large variation in the manner in which CO binds at the surface in the presence of each of the cations during eCO_2_R.

### The CO potential dependent stretching frequency changes as the cation is changed

**Fig. 4:**
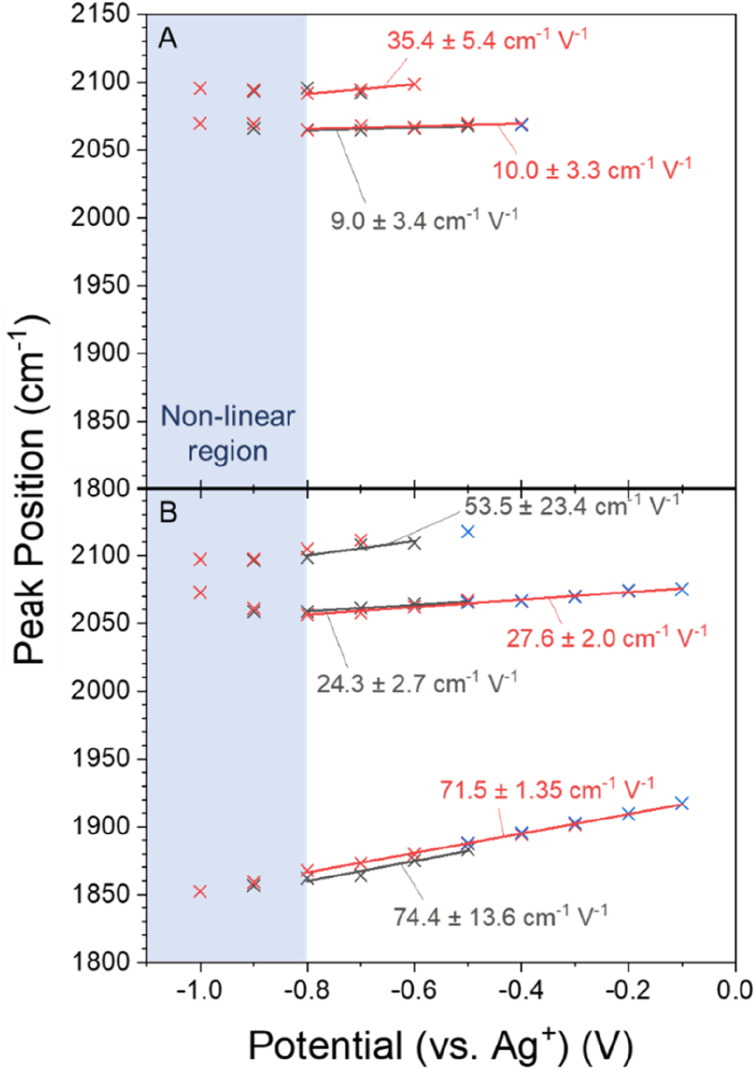
VSFG measured *ν*(CO) on Cu as a function of applied potential with CO_2_ purged (a) 0.5 M NaHCO_3_ and (b) CsHCO_3_ in the −0.5 → −1.0 V (black) and −1.0 → −0.1 V (red) scan directions. Tuning rates in the potential range −0.1 to −0.8 V are annotated and the region in which the frequency tuning rates become non-linear is shaded in blue. Blue data points (crosses) are *ν*(CO) values when the potential is stepped back between −0.1 to −0.5 at the end of the experiment.

Variations in local electric field as the cation is changed have been proposed to be a significant factor in controlling eCO_2_R activity and these may be a factor behind the potential and cation dependence of the CO binding site observed in Fig. 2. Potential dependent *ν*(CO) frequency changes are often used as an indicator of local electric field strength and these are shown in Fig. 4.^57^ The deviation from linearity in the potential dependent frequency tuning < −0.8 V (shaded blue in Fig. 4) demonstrates that directly equating potential dependent frequency tuning to electric field effects is an oversimplification. Consequently, tuning rates here are determined from linear fits of the data between −0.1 and −0.8 V, where the frequency tuning is relatively linear, and only in cases in which there are 3 or more data points so that an error can be calculated. Tuning rates are annotated on the Fig. 4 for the −0.5 to −0.8 V cathodic and the −0.8 to −0.1 anodic scans. A full set of all tuning rates calculated in CO_2_ purged electrolytes can be found in Fig. S8.†

The VSFG experiment in CO_2_ purged CsHCO_3_ shows a tuning rate of 24.3 (±2.7) cm^−1^ V^−1^ for the *ν*(CO_atop(terrace)_) site when the potential is scanned from −0.5 to −0.8 V. The *ν*(CO_bridge_) mode tunes at a higher rate of 74.4 (±13.6) cm^−1^ V^−1^. The difference in both absolute frequency and potential dependant frequency between the CO_bridge_ and CO_atop_ sites can be explained by the increase in backdonation to π* antibonding orbitals as the CO adsorbate coordinates to more metal atoms.^58^ The tuning rate of the *ν*(CO_atop(terrace)_) site is less than half in NaHCO_3_ (9.0 (±3.4) cm^−1^ V^−1^, Fig. 4A and S8b†) compared to CsHCO_3_. Tuning rates for the *ν*(CO_atop(defect)_) site are more difficult to compare; the number of data points in the linear region are not enough to extract associated errors in the case of the anodic scan in CsHCO_3_ and the cathodic scan in NaHCO_3_. Tentative comparison of the tuning rates in opposite scan directions shows a significantly higher rate in the presence of Cs^+^; however, the uncertainty in the linear regression means conclusions cannot be drawn regarding *ν*(CO_atop(defect)_). The potential dependent tuning rates for CO on Cu surfaces measured here fall within the distribution reported. The VSFG measured decrease in tuning rate for Na^+^ and Cs^+^ is in line with past reports, where it has been shown to vary in the order Na^+^ < K^+^ < Cs^+^.^20^

### The cation induced change in CO binding mode correlates with *ex situ* measured changes in electrode morphology due to eCO_2_R

**Fig. 5:**
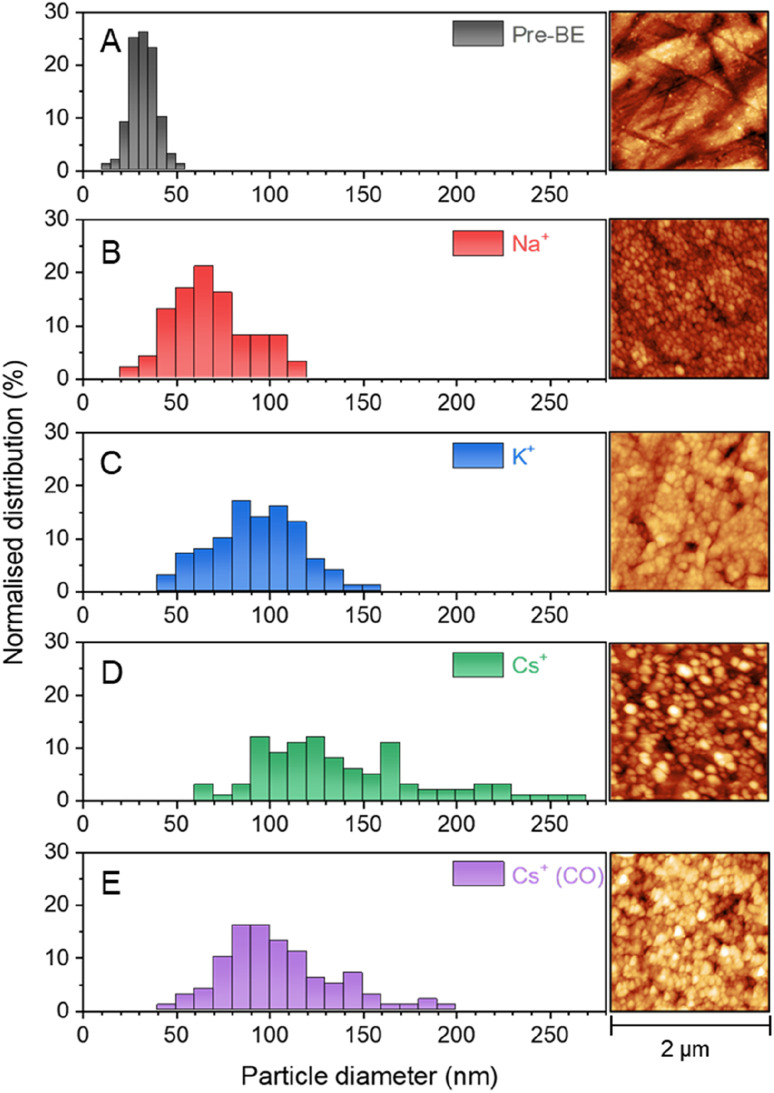
AFM images showing surface morphology of the Cu electrode surface (a) before bulk electrolysis, after bulk electrolysis in CO_2_ purged 0.5 M (b) NaHCO_3_, (c) KHCO_3_ and (d) CsHCO_3_ and also in (e) CO purged 0.5 M CsHCO_3_. Image analysis methods are described in the Experimental section.

VSFG measurements show that the different cations lead to the population of a different set of surface sites by CO during eCO_2_R on Cu. It is known that eCO_2_R can lead to both reversible (with applied potential) and irreversible restructuring of the Cu surface, which would modify available sites for CO binding.^27,59,60^ To assess cation dependent surface structuring, atomic force microscopy (AFM) was used to study the Cu surfaces after cycling through the potential step programme used for spectroelectrochemistry in CO_2_ purged electrolytes, Fig. 5A–D. AFM shows the electrochemical experiments lead to changes in the grain size of Cu surface post-experiment. Interestingly, there is a correlation between cation size and post-electrolysis Cu grain size; the mean grain size increases in the order of pre-bulk electrolysis (32.4 ± 7.0 nm) < Na^+^ (68.7 ± 21.4 nm) < K^+^ (93.3 ± 23.3 nm) < Cs^+^ (139.5 ± 44.1 nm). The AFM map post-electrolysis also has the widest distribution of grain size with Cs^+^ present, indicating particularly high levels of surface reconstruction using this cation. As the CO_bridge_ site is present in CsHCO_3_ when purging with CO_2_ but not CO, AFM images have been recorded of the Cu surface after bulk electrolysis in CO purged CsHCO_3_, Fig. 5E. Similar reconstruction occurs; however the average particle size is lower for the CO purged system (104.3 ± 29.9 nm), closer to that of the CO_2_ purged KHCO_3_ system. We note that it is important to take caution when interpreting *ex situ* characterisation of Cu surfaces as they are not true representations of surfaces under *operando* conditions. *In situ* TEM shows that metallic Cu structures formed under operating conditions are rapidly changed upon exposure to air and subsequent Cu_2_O formation.^61^ Nonetheless, the clear difference in *ex situ* morphology post-electrolysis is indicative of differences in structure *in situ* depending on the cation used offering a rationale for the cation dependence of CO binding site measured *in situ* by VSFG spectroscopy.

VSFG spectroscopy allows us to explore the potential dependence (and stability) of the atop binding modes in the presence of Na^+^ and Cs^+^, Fig. 1. Overall, the potential dependent profiles of the CO_atop(terrace)_ and CO_atop(defect)_*I*_VSFG_ response are similar, with the CO_atop(defect)_ increasing at potentials negative of −0.7 V. The slight decreases in *I*_VSFG_ for CO_atop(defect)_ at the most negative potentials studied (−1.0 V) in the presence of Na^+^, but not Cs^+^, indicate that Cs^+^ leads to stabilisation of CO binding at the defect site and past studies have also proposed that this may occur.^9,11,23^ The potential dependent tuning rates of the CO stretching modes need to be interpreted with caution. Deviations in the linear applied potential *versus* CO frequency relationship are observed at catalytic potentials (<−0.8 V) which is reasonable given that we anticipate both changes in the local environment (electrode structure), as indicated by the post-experiment AFM studies, and changes in CO concentration which will impact on dipolar coupling. The excellent agreement between the anodic and cathodic tuning rates in each electrolyte (Fig. 4), despite clear differences in *I*_VSFG_ observed (Fig. 2), suggests the effects of dipolar coupling in the −0.1 to −0.8 V potential range in our experiments is minimal. It is striking that there is a significant increase in tuning rate of the *ν*(CO_atop(terrace)_) mode in the presence of Cs^+^ compared to Na^+^ which is indicative of an increase in local field strength at the Cu electrode in the presence of CO which will modify Cu–CO stabilities.

However the most striking result is that the nature of the CO binding site is strongly dependent on the cation used. With Na^+^ CO is bound atop at both terrace and defect sites. With K^+^ we propose that a more disordered surface with multiple sites occupied by CO occurs leading to no clear VSFG modes. When Cs^+^ is used, which is widely reported to give the highest levels of eCO_2_R activity,^11–17^ there is a build-up of CO bound at bridge sites on the Cu surface which is not seen with other cations. The CO_bridge_ site is considered electrochemically inactive^33^ making it striking that it is observed using Cs^+^, but not Na^+^ which is usually less active for eCO_2_R. One possible cause of the apparent contradictory results is misassignment of the CO_bridge_ mode. For example, metal impurities can lead to different M–CO modes being present and these have been proposed to be the cause of erroneous assignments in SEIRAS studies.^62^ Common causes of metal ions in the electrochemical cell, such as counter electrode dissolution and impurities from the supporting electrolyte itself, are addressed by the use of carbon counter electrodes and a thorough pre-electrolysis of the electrolyte solution (see Experimental section). Furthermore, observation that the *ν*(CO) at ∼1887 cm^−1^ on Cu is not present during spectroelectrochemical studies of CsHCO_3_ purged with CO, but that it is present during studies of the same electrolyte when CO_2_ purged, supports assignment to an intermediate formed during eCO_2_R on Cu, namely multiply (bridge) bound CO.^30,33,34,38,39,41,47^

Although electrochemically inactive at the potentials studied here, the presence of bridge bound CO may still enhance eCO_2_R activity for C_2+_ products and a number of studies propose that C–C coupling is facilitated by bridge sites during eCO_2_R, with calculations revealing that energetic barriers are lowest for coupling between CO_atop_ and CO_bridge_ sites.^30,34,36,37,63^ In fact, a recent study reports C_2_ FE over 90% at industrially relevant current densities and attributes this catalytic behaviour to the stable ratio of CO_atop_/CO_bridge_ sites, observed *via* Raman spectroscopy.^34^ The observation by VSFG spectroscopy that CO_bridge_ is formed with CsHCO_3_, an electrolyte with an increased C_2_/C_1_ product ratio when compared to K^+^ and Na^+^,^11–17^ strongly supports the involvement of the bridging site in CO–CO coupling.

Potential and pH induced surface reconstruction has been reported to trigger formation of the bridge binding site, with a high pH being particularly beneficial.^33^ Cu surface restructuring is also sensitive to a range of other factors including the initial presence of a surface oxide,^64^ local CO concentration/surface CO coverage^27,48^ and the nature of the electrolyte anion.^38,65^ The presence and nature of alkali metal cations has also been proposed to be a controlling factor^18^ with one study postulating that increased activity for C–C bond formation using Cs^+^ is due to electrolyte induced modification of the Cu surface.^66^ It is therefore striking that the detection of the CO_bridge_ mode *in situ* correlates with increased *ex situ* measured changes in electrode morphology post-electrolysis when Cs^+^ is used as the electrolyte during eCO_2_R.

## Conclusions

VSFG spectroscopy enables the study of a pc-Cu electrode without the need for surface modification/nano-structuring of the electrode which is often applied to linear vibrational spectroscopies to obtain suitable levels of surface sensitivity. There is increasing evidence that the morphology of the electrode can have a profound effect on catalytic activity making it important that spectroscopic studies are carried out on the most commonly employed electrode structures. Past VSFG studies have identified *ν*(C–H) but been unable to identify the important surface bound CO on pc-Cu; here we identify that the detection of CO at the pc-Cu depends on the choice of cation and specific reaction conditions. We measure high levels of CO_bridge_ by VSFG when a Cs^+^ electrolyte is used. We find that there is a correlation between *ex situ* post electrolysis measurements of electrode morphology, which shows that the degree of restructuring is cation dependent, and the presence of the CO_bridge_ intermediates as measured by VSFG spectroscopy. These results suggest that a high level of bridge site formation is related to, or facilitated by, the Cu restructuring that happens as a result of the use of the Cs^+^ cations in the supporting electrolyte. Recent reports have indicated that multiple (bridge) bound CO may be electrochemically inert^33^ but this work builds on the emerging evidence that CO_bridge_ sites are a key intermediate in the CO–CO coupling step that is required for C_2+_ formation during eCO_2_R.^30,34,36,37,63^

## Experimental section

### VSFG cell preparation

All VSFG experiments were performed in a custom spectroelectrochemical “cross-cell”. The cross cell and all components were kept in Milli-Q water between experiments to avoid contamination by organics in the air, with additional thorough rinsing before assembly. The 3 mm diameter Cu (Alvatek) working electrode was mechanically polished for 10 minutes using 1.0 μm then for a further 10 minutes using 0.05 μm alumina suspension. The electrode was rinsed thoroughly and sonicated in Milli-Q water between each polish. The working electrode was then secured into the cross cell with a Ag wire pseudo reference electrode (sanded, rinsed and sonicated before each experiment) and a glassy-carbon counter electrode (mechanically polished before each experiment). Electrolytes used were 0.5 M KHCO_3_ (99.5%, Merck), NaHCO_3_ (99.7%, Fisher) or CsHCO_3_. Due to issues regarding impurities when using commercial CsHCO_3_, CsHCO_3_ was prepared from CsOH (99.95%, Merck) by purging with CO_2_ (99.995%, BOC) for 1 hour, verified by a pH change from 13.6 to 7.4. All electrolytes were pre-electrolysed at −0.025 mA cm^−2^ with Ti plates overnight prior to use to remove any metal impurities. Electrolytes were purged with CO_2_ for 20 minutes in a sealed vial before being cannula-transferred into the cross cell. For the CO purged controls, the electrolytes were first degassed with N_2_ for 20 min before a 1 min purge with CO (99.97%, BOC). Working electrodes were pressed up against the 2 mm thick CaF_2_ window after electrolyte transfer.

### VSFG apparatus

A diagram of the VSFG setup is provided in Fig. S1.† The VSFG experiments were performed using a newly constructed spectrometer. 90% of the output PHAROS-PH1-SP (Light Conversion, 1030 nm, 10 kHz, 10 W, 170 fs pulse duration) laser system is used to generate both the nIR and mIR laser pulses required for the VSFG experiments. 1 W of the output is used to generate a time asymmetric narrow-band nIR pulse (1030 nm, 10 kHz, ∼1.5 ps, ∼13 cm^−1^ linewidth) *via* an etalon (SLS Optics), which is directed to the sample through a half-wave plate (Thorlabs, WPH10M-1030) and polariser (Thorlabs, LPVIS050-MP2) to rotate the light to a horizontal polarisation, and p-polarised w.r.t refection from the sample, at an angle of incidence of ∼45°. A 950 nm long pass filter (Thorlabs, FEL0950) is also used to filter out second harmonic 515 nm light generated within the halfwave plate. This nIR pulse is focused by a 30 cm lens (Thorlabs LB1779-B), with the sample placed ∼25 cm away from this lens, resulting in an approximate beam diameter at the sample of ∼400 μm (1/*e*^2^ diameter) with a power of ∼20 mW. 8 W of the laser output is used in an IR OPA (Light Conversion, Orpheus-One-HE) to generate the broadband IR beam which can be tuned across the frequency range of interest (10 kHz, 170 fs pulse duration and ∼150 cm^−1^ @ 1900 cm^−1^). The mIR output passes through a twisted periscope to switch the polarisation from vertical to the desired horizontal polarisation. The purity of the polarisation is checked using a polariser (Thorlabs, LPMIR050-MP2) which is removed from the beam path prior to VSFG measurement. The mIR beam is focussed onto the sample, with an approximate beam diameter of 300 μm using a Au parabolic mirror (Thorlabs, MPD249H-M01) at an angle of incidence of ∼50° and p-polarisation w.r.t reflection from the sample, with a power of ∼20 mW. A gas-purge generator removes H_2_O, CO_2_ and other contaminants in the air from the IR beam path. Resulting p-polarised VSFG light is directed through 950 (Thorlabs, FES0950) and 900 nm (Thorlabs, FES0900) short pass filters to remove the 1030 nm nIR beam. The beam goes through another polariser (Thorlabs, LPVIS050-MP2) before being focussed using a 15 mm focal length lens (Thorlabs, LA1540-B) through 150 μm slits and into the spectrograph (Andor, Kymera), and is detected on a CCD camera (Andor, iDus416). nIR/mIR delay is introduced using a linear stage (ThorLabs, LTS300C) on the nIR beam path. The spectrograph was calibrated using Ne spectral lines and the rovibrational P, Q and R CO_2_ branches.

### Spectroelectrochemical measurements

Laser beams were always initially aligned off a Au mirror to allow comparison to an IR profile on a clean surface. The potential was stepped from −0.5 → −1.0 → −0.1 → −0.5 V on Cu using a potentiostat (PalmSens). Each potential step was held for 150 s, before sweeping at 50 mV s^−1^ to the next step to avoid current spikes when jumping between potentials. After equilibration for 30 s, VSFG spectra were acquired for 120 s. Spectra were collected for nIR/mIR delays of 1000, 670 and 0 fs in that order.

### AFM characterisation

A contact-mode cantilever (Pointprobe®, NanoWorld®, Switzerland) was inserted onto an AFM nose cone (Keysight Technologies, United States). The nose cone was loaded into an atomic force microscope (5500 AFM, Keysight Technologies, United States) and then the laser was aligned to situate the beam spot on the cantilever tip. After approaching the cantilever, images were acquired using Keysight NanoNavigator software (version 1.8.2), morphologies were analysed using Gwyddion (version 2.61) and size distributions were obtained using ImageJ (version 1.53t). In ImageJ the scale of the edge of each image was set to 2 μm and the diameters of a randomly selected set of 100 particles were determined using the measure function.

## Data availability

Underpinning data including multicurve fits are freely available at on the University of Liverpool Research Data Catalogue (https://doi.org/10.17638/datacat.liverpool.ac.uk/2557).

## Author contributions

LCB: investigation, data analysis, writing – original draft. HJ: investigation, data analysis, writing – original draft. AG: conceptualization, methodology, writing – review and editing, supervision. AJC: conceptualization, supervision, writing – review and editing, project administration.

## Conflicts of interest

There are no conflicts to declare.

## Supplementary Material

SC-015-D3SC05295H-s001
