# Supplementary material for: Studying the cation dependence of CO2 reduction intermediates at Cu by in situ VSFG spectroscopy
Source: Chem Sci. 2024 Jan 17;15(8):2889–97. doi: 10.1039/d3sc05295h (PMC10882457; doi:10.1039/d3sc05295h)
Supplement: SC-015-D3SC05295H-s001 [file SC-015-D3SC05295H-s001.pdf]

Electronic Supplementary Material (ESI) for Chemical Science.
This journal is © The Royal Society of Chemistry 2024

Supporting information

Studying the cation dependence of CO_2 reduction intermediates at Cu by in-situ VSFG spectroscopy

Liam C. Banerji,[1] Hansaem Jang,[1] Adrian M. Gardner,[1,2] Alexander J. Cowan[1*]

1. Department of Chemistry and Stephenson Institute for Renewable Energy, University of Liverpool, Liverpool UK

2. Early Career Laser Laboratory, University of Liverpool, Liverpool UK

* acowan@liverpool.ac.uk

1. VSFG Experimental setup

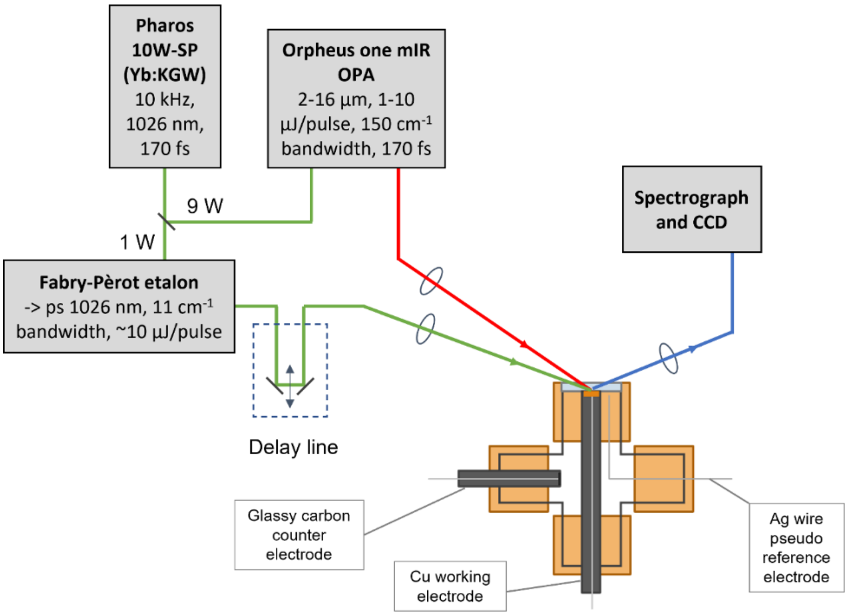

Figure S1: *Experimental setup for VSFG experiment, beam paths of mIR (red), nIR (green) and VSFG (blue)*

Supplementary note 1: Choice of reference electrode

A pseudo-Ag wire reference electrode is used for the spectroelectrochemical experiments in this study. The Ag/AgCl reference electrode is commonly employed in electrochemical CO_2 reduction (eCO_2R) studies, however this introduces the risk of Cl^- contamination in the cell. As vibrational sum frequency generation (VSFG) is a highly sensitive technique, great care was taken to avoid introducing contamination into the cell, so Cl^- was eliminated through the use of the Ag wire alone. There is the further possibility of Ag^+ contamination, which could then adsorb onto the Cu working electrode under reduction conditions, however the vibrational frequency for CO (ν(CO)) adsorbed on Ag has been shown to be at significantly different positions compared to the ν(CO) modes observed in this work.[1]

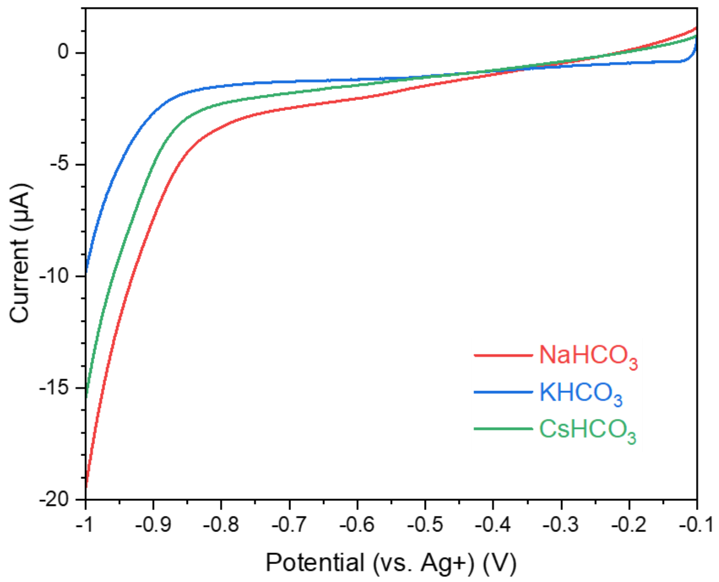

Figure S2: *LSV of the Cu electrode in the spectroelectrochemical cell in each of the electrolytes across the potential range used in the study*

Supplementary note 2 – non-resonant SFG response suppression

Each VSFG experiment was repeated under 3 different delay conditions: 0, 670 and 1000 fs. When there is no temporal delay in the overlap of the near infrared (nIR) and mid infrared (mIR) pulses (i.e. t = 0 fs), the VSFG spectra exhibit features referred to from here onwards as resonant and non-resonant (NR). The NR feature is reflective of the mIR spectral profile, upconverted by the frequency of the nIR pulse. Any resonant vibrational modes that exist

within the mIR spectral envelope appear as superimposed features, with intensities dependant on their phase relative to the NR (and each other if they are close in energy). As the lifetime of the mIR pulse is much shorter than the lifetime of the resonant modes, introducing a delay between the mIR and nIR pulses allows for suppression of the NR feature leaving only the resonant vibrational modes of interest in the spectra.

The interactions between the resonant modes and the supressed NR are important to consider when analysing the data. For example, at certain delays vibrational modes will be out of phase with the NR pulse reducing their apparent intensity, causing them to be unobservable. Over limited delay ranges, interactions with the NR can also lead to artificial shifts in frequency and misleading peak shapes of the vibrational modes, causing misinterpretation of peak positions. For these reasons, each electrode/cation combination is tested at the three nIR/mIR delays shown in Figures S3–S8: a) 0 fs, allowing identification of the modes present with no quantum beating effects whilst giving the shape of the underlying mIR profile, b) 670 fs, adequately supressing the NR signal to give clear identification of the dominant resonant modes and c) 1 ps, high suppression giving poor signal-to-noise. 1 ps delays clearly showed suppression of the NR signal, but the resonant modes have decreased to a level that makes detection challenging. For this reason, the 670 fs scans are presented. Scans are collected in the order of 1 ps, 670 fs then 0 fs. CO is generated at the surface in the first scan, explaining the presence of adsorbed CO on the Cu surface more positive than the eCO_2R onset potential (Figure S1) in the 670 fs and 0 fs scans.

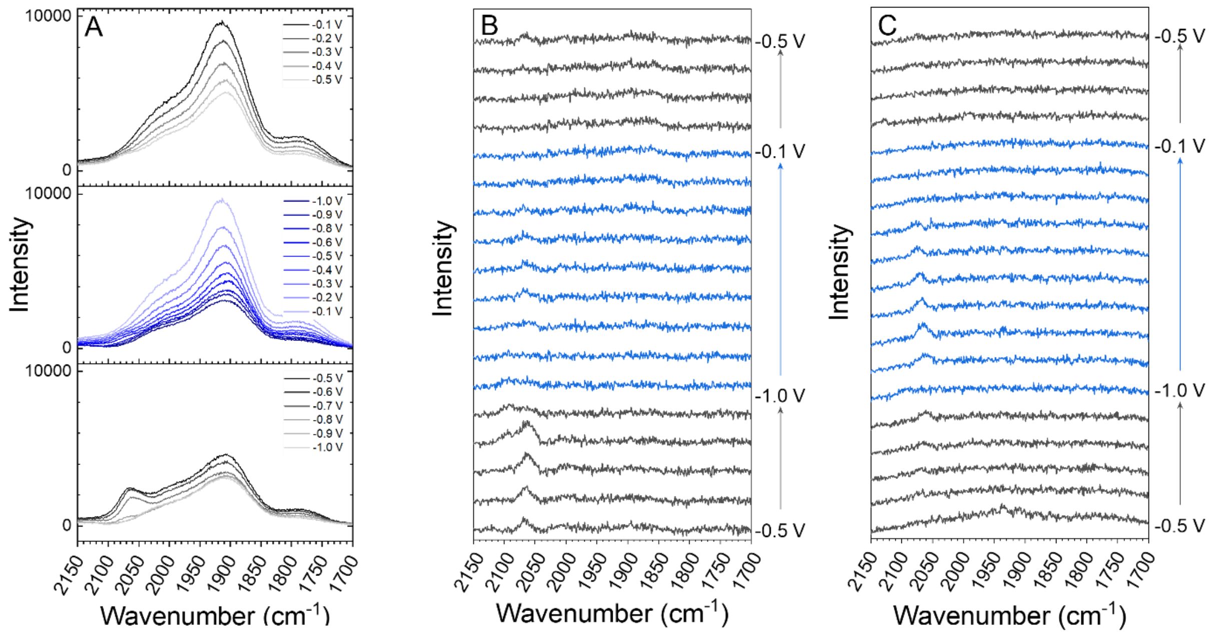

Figure S3: *VSFG spectra of CO at Cu during eCO₂R in 0.5 M NaHCO₃ with a) 0 fs, b) 670 fs and c) 1000 fs nIR/mIR delay*

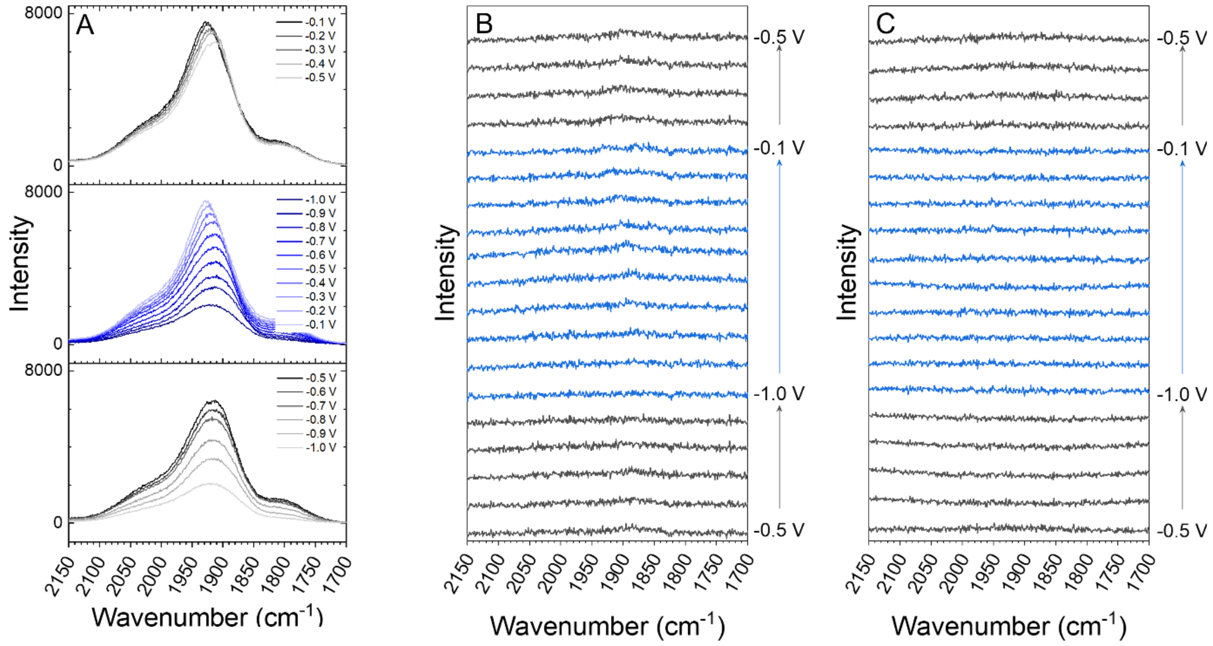

Figure S4: *VSFG spectra of CO at Cu during eCO₂R in 0.5 M KHCO₃ with a) 0 fs, b) 670 fs and c) 1000 fs nIR/mIR delay*

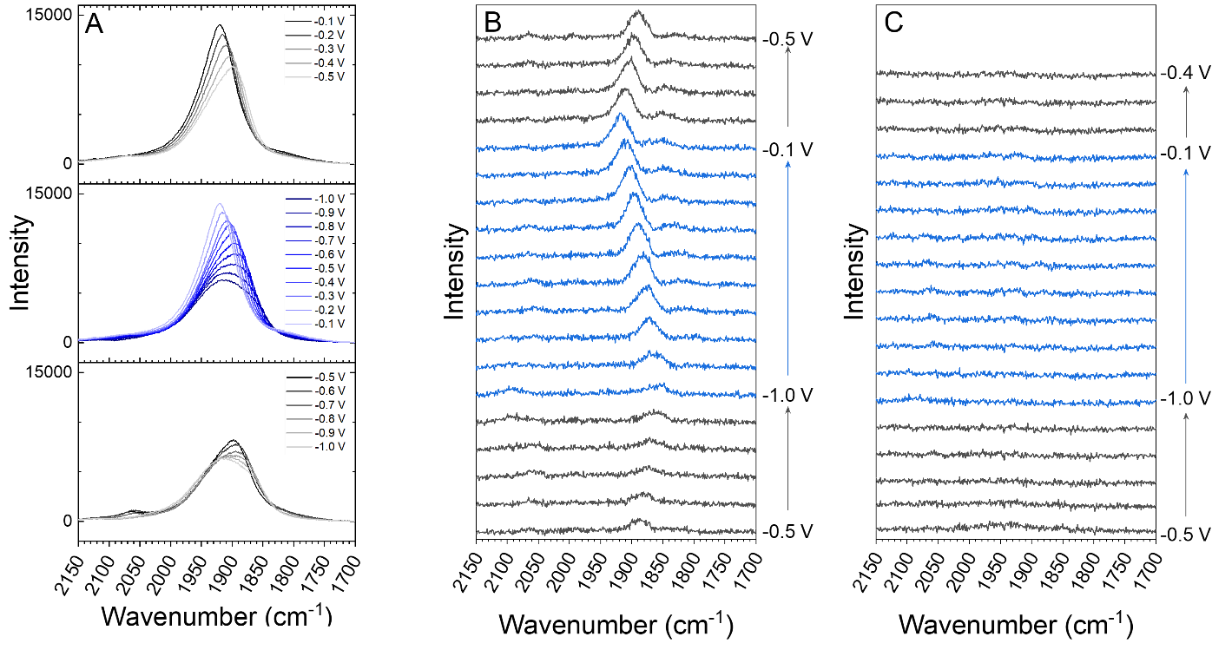

Figure S5: *VSFG spectra of CO at Cu during eCO₂R in 0.5 M CsHCO₃ with a) 0 fs, b) 670 fs and c) 1000 fs nIR/mIR delay*

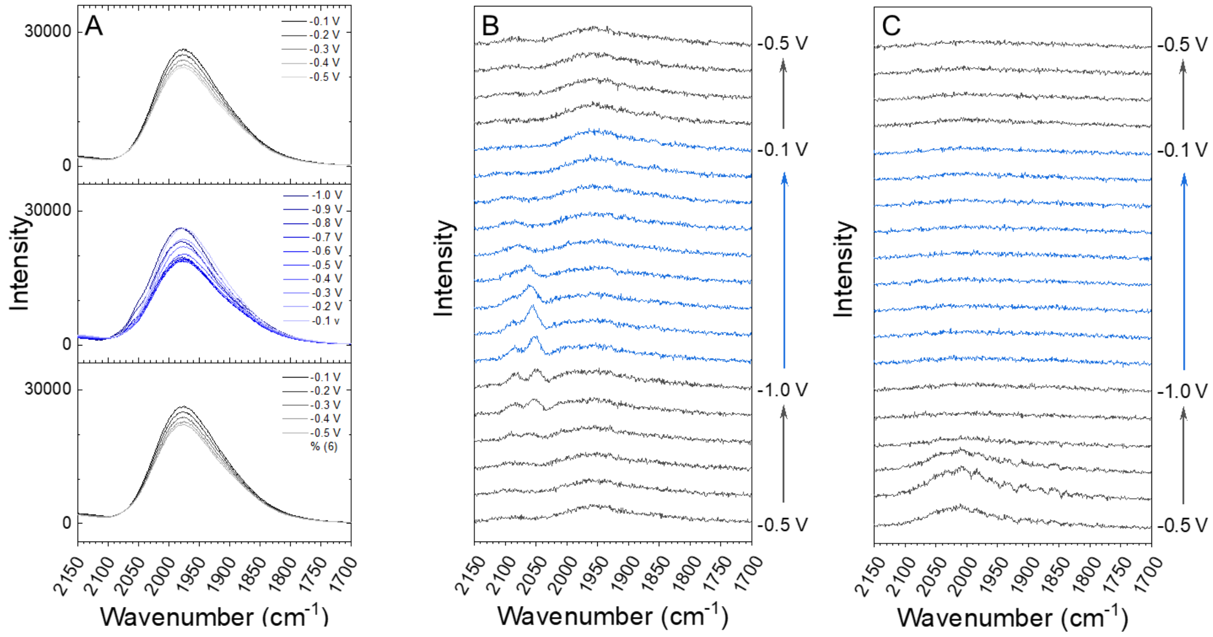

Figure S6: *VSFG spectra of CO at Cu during eCOR in 0.5 M CsHCO₃ with a) 0 fs, b) 670 fs and c) 1000 fs nIR/mIR delay*

Supplementary note 3 – normalisation of VSFG spectra

The intensity of a peak at a certain frequency observed in the VSFG spectra is dependent on the relative intensity of the mIR pulse at that frequency. Therefore, it is necessary to normalise the spectra to the mIR spectral shape in order to extract accurate information on the peak intensities. Figure S5 shows each of the spectra collected at a 670 fs nIR/mIR delay (shown in the main text) divided by the spectra at 0 fs before the beginning of the first experiment, i.e. the NR with no superimposed resonant features. This is how the data is presented in the main text. Normalisation of the data in this way can lead to artefacts towards the edges of the NR window as the intensity drops to very low levels and is responsible for the variation in the signal to noise ratio across the spectral region of interest.

In the case of the CO purged $CsHCO_3$ system (Figure 3 in the main text), the normalised spectra exhibit a broad feature between ca. 2050 to 2150 cm^{-1}. This is suspected to be due to supercontinuum generation in the CaF_2 window of the spectroelectrochemical cell owing to slight differences in cell position in this experiment cf. those in CO_2 purged solutions. Due to its interference with the CO_{atop} peaks (both terrace and defect), it is necessary to remove this to analyse the peak positions and areas. This has been done by using the first −0.5 V spectrum, which has no observable CO_{atop} modes, Figure S7a, as a background for subtraction. The resulting spectra and peak positions are given in Figure S7.

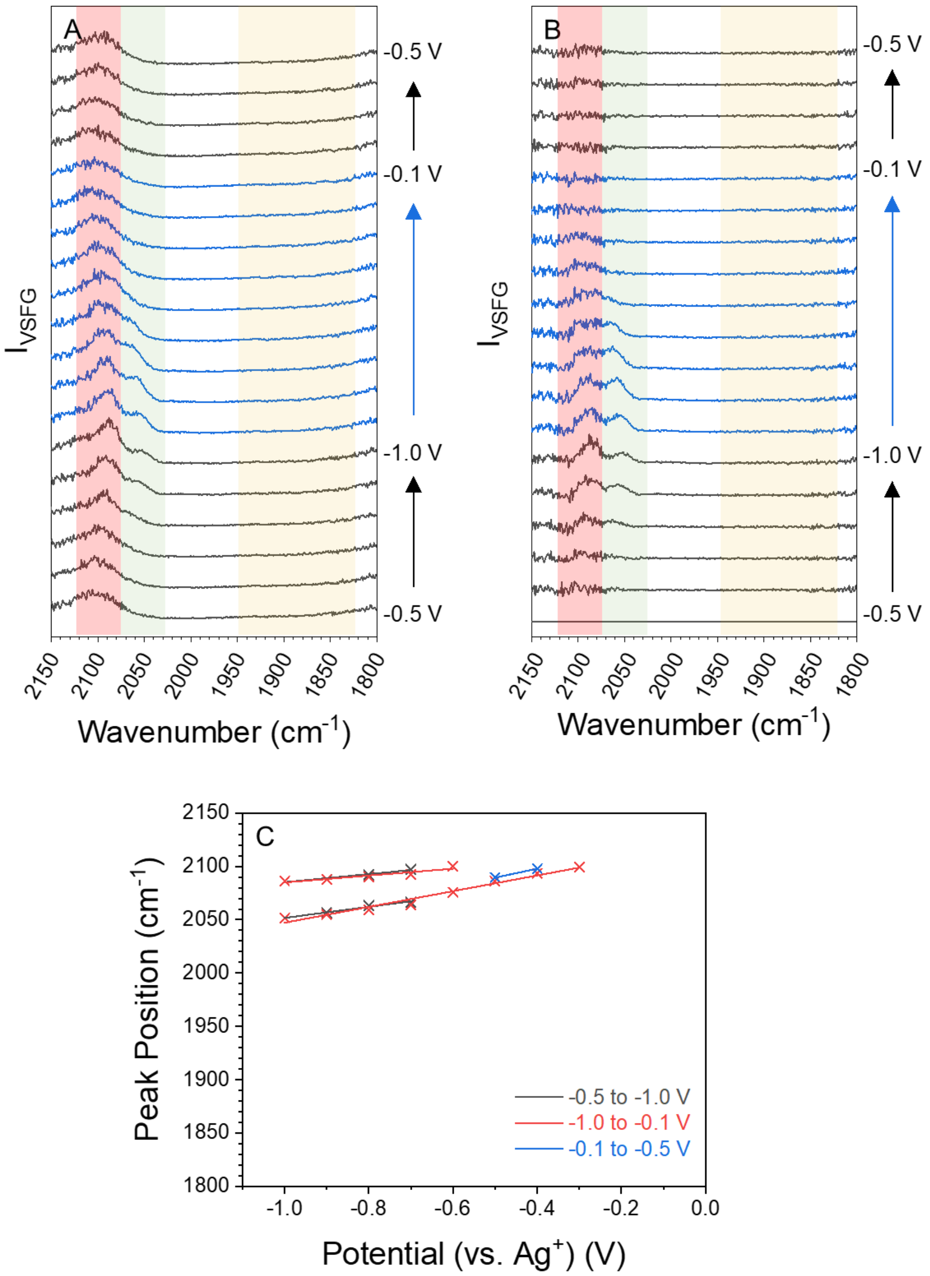

Figure S7: *a) mIR normalised VSFG spectra for experiments in CO purged* $CsHCO_3$*, b) the same spectra after a background subtraction using the first −0.5 V spectrum to remove interference from super continuum generation (presented in the main text), c) the peak positions extracted from the fitting of the background subtracted spectra.*

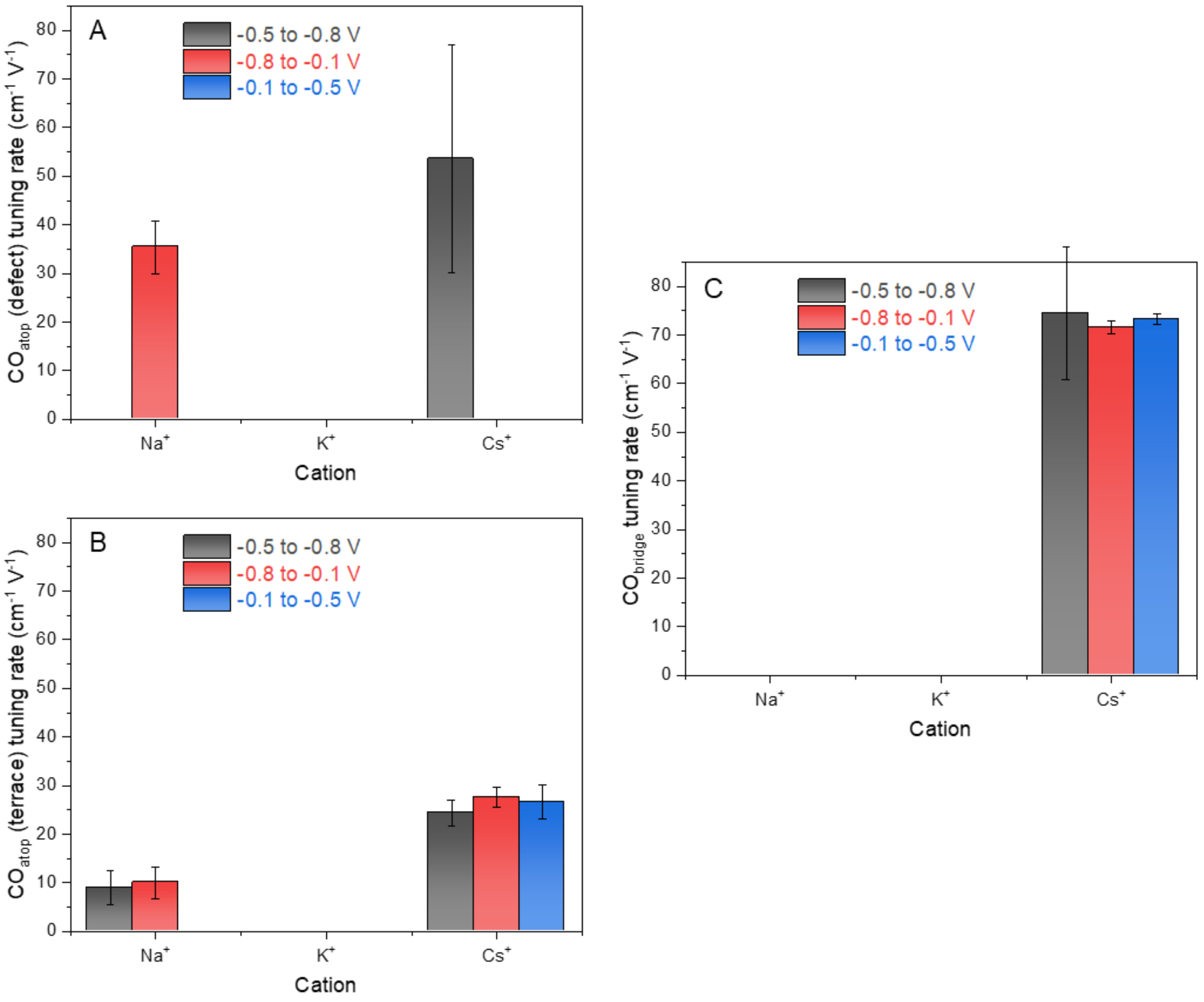

Figure S8: *Potential dependent frequency tuning rates in each CO_2 purged electrolyte system for a) CO_{atop} (defect), b) CO_{atop} (terrace) and c) CO_{bridge} sites.*

Potential dependent frequency tuning rates for CO_2 purged electrolytes, calculated using the peak positions determined by Lorentzian peak fitting, are provided in Figures S8. Absence of columns indicates that peaks are observed at either one or none of the potential steps in the relevant window, making it impossible to calculate a tuning rate, or that peaks are observed across only two of the potential steps in the relevant window and no error could be calculated by linear regression.

Supplementary note 4 – electrochemical surface area (ECSA)

Electrochemical surface area of the electrode in each of the electrolytes was determined using the double layer capacitance (C_{DL}). −0.5 to −0.6 V is the most non-Faradaic region in the potential window so is chosen for these measurements. Cyclic voltammetry (CV) scans are

conducted in this window at scan rates of 100, 200, 300, 400 and 500 mV s^{-1} for 10 cycles each, then the current is extracted from the final cycle and plotted against the scan rate. The gradient gives C_{DL} which is used to compare the relative ECSA of the Cu surface in each electrolyte. Figure S9 shows the CV and the resulting C_{DL} values.

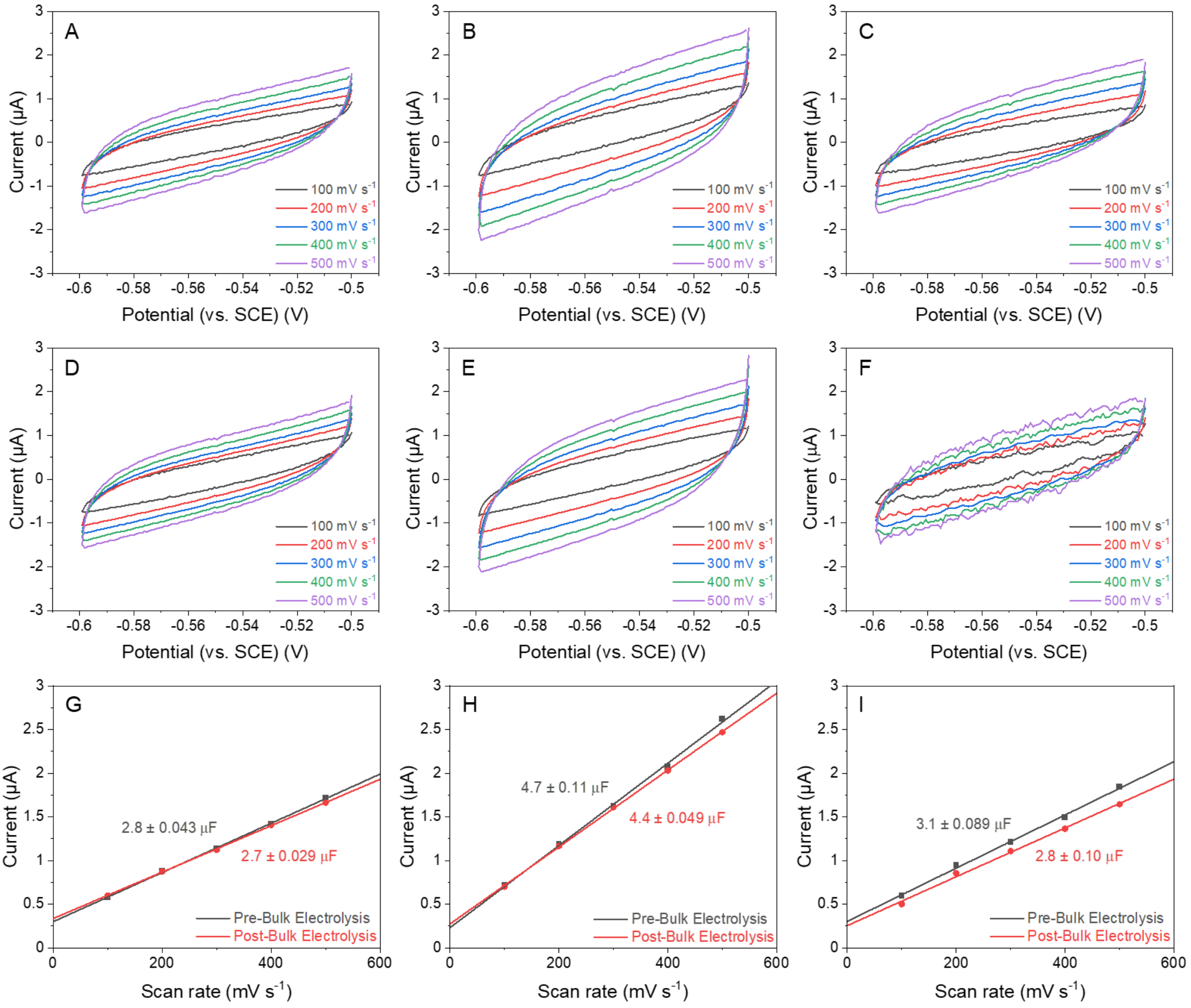

Figure S9: *CV measurements in the non-Faradaic region of Cu pre- (a-c) and post- (d-f) bulk electrolysis in $NaHCO_3$ (left), $KHCO_3$ (middle) and $CsHCO_3$ (right). (g-i) C_{DL} values obtained from plotting the difference in the current at −0.55 V against the scan rate.*

Supplementary note 5 – X-ray photoelectron spectroscopy (XPS)

On Au electrodes, mechanical polishing with aluminium oxide powder can result in adventitious contamination at the electrode surface.[2] To examine the surface contamination under our experimental conditions, we prepared highly pure, Al-free Cu electrodes (Table 1,

Lot No. Y30H024, ThermoFisher Scientific) and identified their elemental composition using X-ray photoelectron spectroscopy (XPS; HarwellXPS, United Kingdom). Prior to the XPS, Cu electrodes underwent different experimental conditions to facilitate comparisons and the details are tabulated in Table 2. As the primary XPS region of Al is 2p (zero-valent Al $2p_{3/2}$ at 72.6 eV with $\Delta = 0.44$ eV)[3,4] which overlaps Cu 3p (zero-valent Cu $3p_{3/2}$ at 74.9 eV with $\Delta = 2.45$ eV),[5,6] we began with fitting Cu 3p peaks so that the rest of the spectrum can be assigned as the contribution from Al 2p identity. The Cu 3p doublet was fitted on the basis of refs [5,6] with a standard Shirley background and a pseudo-Voigt function as summarized in Table 3. This fitting condition gives a good match between an experimental Cu spectrum and the envelope of fitted Cu 3p peaks for Sample 1 (Figure S10a). In this sample, only a single doublet responsible for metallic Cu was identified without other oxidation states or elements. On the other hand, the Cu 3p spectrum for Sample 2 was deconvoluted into two doublets: one assignable for metallic Cu and the other for Cu of higher oxidation states such as CuO (Figure S10b). This indicates that mechanical polishing with alumina modifies the electrode surface, making it more susceptible to oxidation, giving rise to native oxide layer detectable by XPS. Despite the use of alumina for the polishing, any peaks corresponding to Al or Al oxides (oxide Al 2p at 74.6 eV;[3] native oxide Al 2p at 75.6 eV)[3] are not apparent. A similar trend was found in Sample 3 (Figure S10c) – Al-based signals are not detected in the post-electrolysis sample. Interestingly, Cu oxide contribution is more pronounced in the post-electrolysis sample (12.9% of total area) than the pre-electrolysis sample (5.8%). Since Sample 3 was exposed to cathodic reactions during the electrolysis, it seems that the formation of oxide layer is not directly related to the electrolysis process, per se, but could be attributed to a change in surface morphology or other factors caused by the electrolysis. To sum up, it appears that mechanical polishing with alumina powder plays a major role in modifying the surface morphology of the electrodes, with little contribution to adventitious contamination by Al or Al oxides, at least under the experimental conditions used in this study.

Table 1. Concentration of impurities present in the Cu electrode (purity >99.997%)

	Ag	As	Bi	Cd	Fe	Mn	Ni	O	P	Pb	S	Sb	Se	Sn	Te	Zn
ppm	12	0.7	0.8	<0.4	1.5	0.3	1.3	1.5	0.3	<0.7	5.8	<1	<0.6	<0.7	<2	<0.1

Table 2. Sample experimental conditions

	Description	Notes
Sample 1	Pristine Cu (as-received)	Figure S10a
Sample 2	Cu polished with alumina, pre-electrolysis	Figure S10b
Sample 3	Cu polished with alumina, post-electrolysis	Figure S10c

Table 3. Parameters used for Cu 3p fitting

	Cu	Cu oxide
Spin–orbit splitting	2.45 eV	2.45 eV
Lorentzian–Gaussian mixing ratio	0.9	0.6

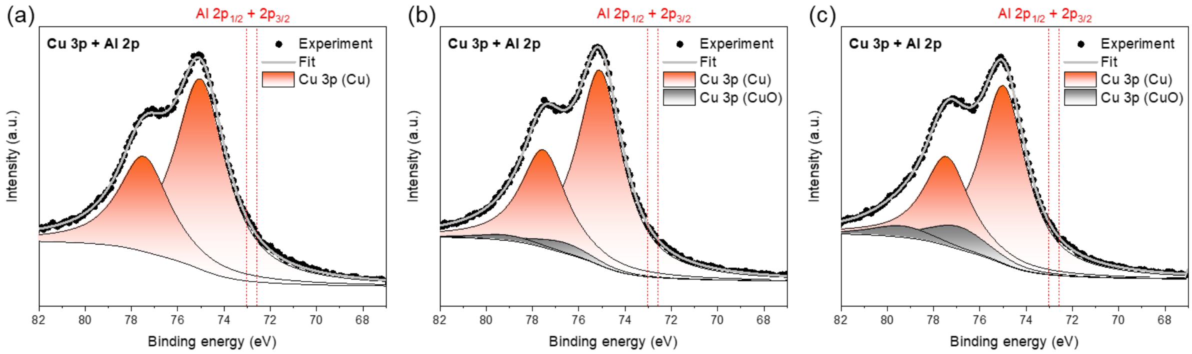

Figure S10: *XPS Co 3p and Al 2p scans for (a) Sample 1, (b) Sample 2, and (c) Sample 3*

Supplementary note 6 – mass transport limitations

In the cell configuration used in the experiments of this study, the surface of the Cu working electrode is pressed up against the CaF_2 window (Figure S1). This is done to limit mIR absorption by water by minimizing the pathlength through the electrolyte. CO_2/CO mass transport will be limited in this thin-layer electrolyte approach; however we note that the electrode surface does not sit exactly parallel to the CaF_2 and there will be regions with a thicker layer of electrolyte. VSFG experiments can be performed in the Kretschmann configuration,[7] in which the electrode is deposited onto the surface of an attenuated total reflection (ATR)

crystal and the beams approach through crystal instead of the electrolyte, however this eliminates the major benefit of using an unmodified commercial polycrystalline Cu electrode instead of a thin film which may exhibit altered electrochemical behaviour.

References

1 Y. Deng and B. S. Yeo, *ACS Catal.*, 2017, **7**, 7873–7889.

2 M. C. O. Monteiro and M. T. M. Koper, *Electrochim. Acta*, 2019, **325**, 134915.

3 https://www.thermofisher.com/uk/en/home/materials-science/learning-center/periodic-table/other-metal/aluminium.html, retrieved on 27 November 2023.

4 NIST X-ray Photoelectron Spectroscopy Database, https://srdata.nist.gov/xps/, retrieved on 27 November 2023, DOI: 10.18434/T4T88K.

5 I. Khalakhan, M. Vorokhta, X. Xie, L. Piliai and I. Matolínová, *J. Electron Spectros. Relat. Phenomena*, 2021, **246**, 147027.

6 R. Hesse, P. Streubel and R. Szargan, *Surf. Interface Anal.*, 2007, **39**, 381–391.

7 S. Wallentine, S. Bandaranayake, S. Biswas and L. R. Baker, *J. Phys. Chem. A*, 2020, **124**, 8057–8064.
